# A Microplate-Based Nonradioactive Protein Synthesis Assay: Application to TRAIL Sensitization by Protein Synthesis Inhibitors

**DOI:** 10.1371/journal.pone.0165192

**Published:** 2016-10-21

**Authors:** Curtis J. Henrich

**Affiliations:** 1 Molecular Targets Laboratory, Center for Cancer Research, National Cancer Institute, Frederick, Maryland, 21702, United States of America; 2 Basic Science Program, Leidos Biomedical Research, Inc., Frederick National Laboratory for Cancer Research, Frederick, Maryland, 21702, United States of America; Indian Institute of Science Education and Research, INDIA

## Abstract

Non-radioactive assays based on incorporation of puromycin into newly synthesized proteins and subsequent detection using anti-puromycin antibodies have been previously reported and well-validated. To develop a moderate- to high-throughput assay, an adaptation is here described wherein cells are puromycin-labeled followed by simultaneously probing puromycin-labeled proteins and a reference protein *in situ*. Detection using a pair of near IR-labeled secondary antibodies (InCell western, ICW format) allows quantitative analysis of protein synthesis in 384-well plates. After optimization, ICW results were compared to western blot analysis using cycloheximide as a model protein synthesis inhibitor and showed comparable results. The method was then applied to several protein synthesis inhibitors and revealed good correlation between potency as protein synthesis inhibitors to their ability to sensitize TRAIL-resistant renal carcinoma cells to TRAIL-induced apoptosis.

## Introduction

Incorporation of puromycin into proteins during translation coupled with subsequent detection using anti-puromycin antibodies has been well-validated in recent years as a method to monitor protein synthesis by recognition of newly synthesized proteins. Applications described in the literature include FACS analysis of synthesis of cell surface proteins [1], visualization of nascent polypeptides in cells and tissues [1–4], detection/quantitation in isolated polysomes [5] and in western blots [1, 3, 6]. Given that puromycin is itself an inhibitor of protein synthesis and to avoid cellular toxicity, sublethal concentrations and short treatment times have been used. When protein synthesis has been quantitated in these examples, it has typically employed fluorescence detection (flow cytometry or fluorescence microscopic localization) and/or western blot analysis. However, in order to accurately quantitate protein synthesis in moderate- to high-throughput applications, a microplate reader-based format would be advantageous.

The In-Cell Western (ICW) technique, an approach to quantitative cell-based protein analysis in 96- and 384-well plates, has become increasingly commonly used for assessment of cell signaling events, particularly protein phosphorylation [7, 8]. ICW is an immunofluorescence approach utilizing near infrared fluorophore-conjugated antibodies to measure individual proteins or to simultaneously measure pairs of proteins in fixed cells. In many signaling applications, antibody pairs recognize total protein and phosphoprotein levels for a kinase or phosphatase substrate protein. Alternatively, quantitation of a target protein may be assessed in comparison to a reference protein for normalization of signals [9, 10]. The latter approach proved to be ideal for application of the ICW technique to microplate-based assessment of protein synthesis in cultured cells. The ICW method is more amenable to greatly increased numbers of repeats of given conditions as well as assessing much more significant numbers of conditions (*e*.*g*., 384 potential experiments per run compared to 12–15 by standard western blot) while also allowing for less subjectivity in quantitation (*e*.*g*., defined wells rather than lanes on a blot).

Use of protein synthesis modulators is of obvious interest in a wide range of biological applications, including regulation of apoptosis in cancer cells. Although use of tumor necrosis factor-related apoptosis-inducing ligand (TRAIL) in cancer therapy has long been an attractive goal given its reported ability to induce apoptosis in cancer cells but not normal cells in a variety of oncologic malignancies, development of TRAIL resistance is common in many types of cancer [11–15]. Inhibition of protein synthesis to reduce levels of cFLIP has been reported as one of a number of cellular processes to target for overcoming resistance of cancer cells to TRAIL-induced apoptosis [15, 16]. In order to further investigate the effects of protein synthesis inhibitors, a moderate- to high-throughput non-radioactive, cell-based protein synthesis assay in microplate format would be desirable. Therefore, such an assay was developed, based on puromycin incorporation into nascent polypeptides and quantitation by the ICW method. This was then used to assess the relationship of protein synthesis inhibition with TRAIL sensitization by 5 protein synthesis inhibitors: anisomycin, cycloheximide, emetine, glaucarubinone, and verrucarin A. Four of these (anisomycin, cycloheximide, emetine, and verrucarin A) have been previously reported to sensitize TRAIL-resistant cells to TRAIL-induced apoptosis [17–20]. Glaucarubinone has not been previously reported as a TRAIL sensitizer, but a related quassinoid has been [21]. In each case, inhibition of protein synthesis (as measured by the ICW assay) correlated with sensitization of TRAIL-resistant cells to TRAIL-induced apoptosis thus demonstrating the utility of the ICW method for addressing an important cellular phenomenon.

## Materials and Methods

### Materials

Unless otherwise noted, general reagents were obtained from Sigma (St. Louis, MO), cell culture reagents and electrophoresis/western blot supplies were from Invitrogen (Carlsbad, CA) and cell culture plasticware from Corning (Corning, NY). Monoclonal mouse anti-puromycin clone 12D10 [1] and rabbit anti-GAPDH (ABS16) were obtained from EMD-Millipore (Billerica, MA). Monoclonal mouse anti-cFLIP clone 7F10 was obtained from Enzo (Farmingdale, NY). Secondary antibodies, Goat anti-rabbit (800) and Goat anti-mouse (680) and blocking buffer were from LiCor (Lincoln, NE). Sources of other reagents: 2,3-Bis(2-methoxy-4-nitro-5-sulfophenyl)-5-[(phenylamino)carbonyl]-2H-tetrazolium hydroxide (XTT; NSC 601519) from the NCI Drug Synthesis and Chemistry Branch; recombinant TRAIL ligand (168 amino acid TNF homologous extracellular domain—Peprotech). Caspase 8 assay kit (CaspaseGlo 8) was obtained from Promega (Madison, WI) and was used according the manufacturer’s directions.

### Cell culture

ACHN renal carcinoma cells were maintained as previously described [22] and seeded into 6-well plates at 10^6^ cells/well for standard western blot assays or into 384-well plates at the indicated concentration for ICW applications. Given the extensive washing required and the fluorescence-based detection and quantitation, black-wall, clear-bottom, polylysine-coated plates were utilized. TRAIL sensitization assays were performed as described [22]. Briefly, ACHN cells were allowed to attach overnight (3500 cells/well, 384-well plates) followed by 4 h with compounds or DMSO. After 24 h additional incubation ± TRAIL, cell survival was estimated byXTT [22]. Cell survival was normalized to vehicle controls.

### Standard western blot analysis

For assay development, cells in 6-well plates were allowed to attach overnight, then were pretreated in the presence or absence of varying concentrations of cycloheximide for 15 min followed by additional incubation in the presence or absence of puromycin (10 μg/ml final) for indicated time periods. Cells were rinsed with PBS then extracted with RIPA buffer supplemented with protease and phosphatase inhibitors (Pierce, Rockford, IL). Gels were loaded with 25 μg/lane and subjected to electrophoretic separation and transfer to PVDF membranes followed by blocking (LiCor Blocking buffer), incubation with anti-puromycin and anti-GAPDH (1:50,000 and 1:10,000 respectively in blocking buffer), and detection with near IR-labeled secondary antibodies. Blots were scanned using the LiCor Odyssey scanner (LiCor Biosciences, Lincoln, NE) and each channel visualized independently or simultaneously. For quantitation, entire lanes in the blot were selected and fluorescence in each channel quantitated using the LiCor software. Control lanes included extracts from cells treated without puromycin.

### ICW assay

For assay development, cell conditions and treatment were the same as described above.

After treatment, cells were fixed by removing medium and adding formaldehyde (1:10 dilution from stock, *i*.*e*. final 3.8% v/v) at 25 μl/well and incubating at room temperature for 20 min followed by washing and permeabilization (5 x 75 μl/well PBS + 0.1% (v/v) Triton X-100). (NOTE: Due to its toxicity, formaldehyde and washes were handled with appropriate personal protective equipment and in a chemical fume hood.) Wells were then blocked (> 1.5 h, room temperature with rocking) with LiCor blocking buffer followed by incubation with primary antibodies in blocking buffer (with rocking; 2 h, room temperature or overnight at 4°C). Based on preliminary results, subsequent experiments used anti-puromycin and anti-GAPDH (1:5000 and 1:1000–1:2500 respectively) premixed in blocking buffer. After extensive washing (5 x 75 μl/well PBS + 0.1% (v/v) Tween-20), wells were incubated with secondary antibodies. Based on preliminary results, subsequent experiments used anti-mouse and anti-rabbit antibodies at 1:5000 each premixed in blocking buffer. Wells were again extensively washed (5 x 75 μl/well PBS + 0.1% (v/v) Tween-20). Final wash was removed and plates were scanned on the Odyssey in “InCell Western” mode capturing relative fluorescence in each channel. In parallel, fluorescence in each channel can be visualized independently or simultaneously using the Odyssey software. Controls included wells with no cells, cells treated in the absence of puromycin, and detection in the absence of primary antibodies (puromycin-treated cells). For further applications, cells were pretreated with inhibitors for 15 min or 4 h followed by processing and quantitation as described above. The same protocol was followed for assessing the levels of cFLIP protein, using mouse anti-cFLIP (1:500) and rabbit anti-GAPDH (1:1000).

### Data capture/data analysis

Raw fluorescence data from the Odyssey software were exported into Excel for analysis. Background was subtracted based on fluorescence in the absence of cells (all negative control values were essentially identical—see S1 File and S1 Fig) and then normalized to untreated (but puromycin-treated) control cells and reported as % of control. IC_50_ values were estimated from dose-response curves using SigmaPlot 4-parameter logistic nonlinear regression analysis (Systat Software). Standard error (se) was calculated using the SigmaPlot software.

## Results

### Development and validation of a quantitative ICW assay

#### Antibody qualification

**Fig 1:**
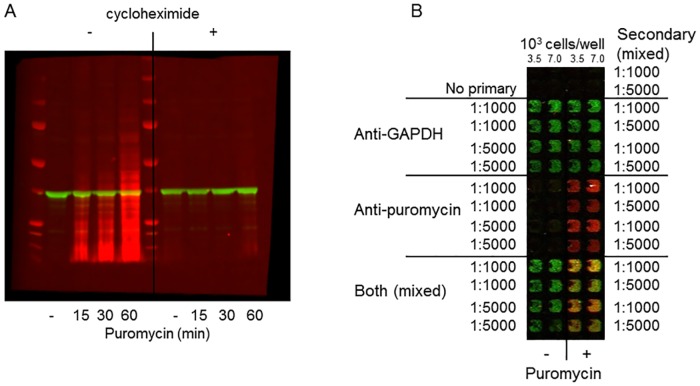
Simultaneous visualization of puromycylated proteins (red) and GAPDH (green). (A) western blot format: ACHN cells were pretreated for 15 min ± cycloheximide (100 μM final) followed by puromycin for the indicated times. After extraction, electrophoretic separation, and transfer (25 μg/lane), detection employed anti-puromycin and anti-GAPDH followed by near IR-fluorophore-labeled secondary antibodies. (B) ICW format: ACHN cells were plated in 384-well plates at the indicated densities and treated ± puromycin (30 min) followed by analysis using the indicated antibody concentrations and visualization.

Since the ICW technique detects and quantitates targets in a cellular environment in multi-well plates rather than after electrophoretic separation, it is necessary to employ highly specific antibodies. For these studies, a mouse anti-puromycin monoclonal antibody was paired with a rabbit anti-GAPDH as a cellular control. Detection employed anti-mouse and anti-rabbit secondary antibodies each labeled with a different near IR fluorophore. Fluorescence in turn was assessed by simultaneous quantitation of both signals using the LiCor Odyssey scanner designed for this purpose. To confirm that the antibodies and quantitation system cleanly and accurately detected their targets, puromycin labeling and GAPDH control were first assessed in a standard western blot format. Puromycin labeling of cellular protein followed previous publications (10 μg/ml for 0–60 min, *i*.*e*. sublethal conditions) and a well-characterized, now commercially-available anti-puromycin antibody (12D10 mouse monoclonal [1]) was chosen for detection of puromycin-labeled protein. Fig 1A (showing simultaneous visualization of signals in both channels) demonstrates lack of cross reactivity and puromycin dependence of the puromycin antibody (red signal) and the GAPDH antibody (green signal), except at the longest puromycin labeling time. Furthermore, the puromycin signal, but not the GAPDH signal, was eliminated by inhibition of protein synthesis by a 15 min cycloheximide pretreatment. These results demonstrate global labeling of newly synthesized protein (*i*.*e*., smear of puromycin signal down the blot) similar to previous applications of the same technology in western blot applications [1] and the GAPDH signal detects only a single band. The signals can be quantitated by selecting sample lanes and recording total fluorescence in each channel (S2 Fig and S1 Table).

#### ICW

**Fig 2:**
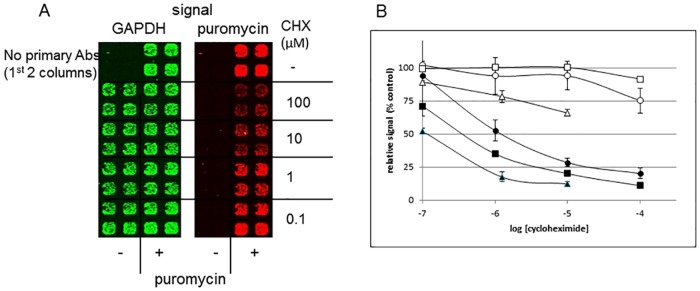
Quantitation of protein synthesis and inhibition by cycloheximide. (A) ICW format: ACHN cells (3000 cells/well) were treated for 15 min ± cycloheximide followed by 30 min ± puromycin. GAPDH (green) and puromycin (red) signals were visualized as described in the text. (B) Puromycin and GAPDH signals from ICW (data from panel A, represented by circles) and western blot (see S2 Fig—represented by squares). Cells were pretreated with 0–100 μM cycloheximide (15 min) followed by puromycin (30 min) and quantitation. In a separate ICW experiment, cells were treated with cycloheximide for 4 h then puromycin (30 min) and quantitation (represented by triangles). Black symbols represent puromycin signal, open GAPDH. All values were normalized to vehicle controls. Error bars represent sd (n = 4).

Based on the standard western blot results, a 30 min puromycin labeling time was chosen and was used for development of a microwell-based method. In order to define appropriate ICW conditions, effects of key variables (cell number and antibody dilutions) were assessed (Fig 1B). Once again, in the absence of puromycin treatment, there was no anti-puromycin signal, nor was there carry-over between the two signals. Thus, the signals are very clean allowing for easy determination of background signal(s). Measurement of total fluorescence in each channel in each well allowed for quantitation of each signal. There were no differences between background signals defined by wells containing no cells or wells incubated with no primary antibodies, or for the red signal cells treated in the absence of puromycin (S1 Fig). The results in Fig 1B allowed for identification of antibody dilutions as well as confirming appropriateness of the puromycin treatment conditions. For routine assay in the ICW format, anti-puromycin and anti-GAPDH antibodies were used at 1:5000 and 1:1000 respectively and secondary antibodies at 1:5000 each. The cell number (3500/well) used in subsequent experiments was chosen to match conditions previously used in a high-throughput screening campaign with this cell line [22]. In order to further validate the results, the ICW technique was applied to cycloheximide-treated cells (Fig 2). Panel A shows an image of an ICW plate. When quantitated, the results were compared to parallel results from a standard western blot experiment in which quantitation was based on entire lanes of the blot (Fig 2B and S2 Fig). There is a very close correlation between quantitative results obtained by each method. In each case, the GAPDH signal was not significantly changed as a result of cycloheximide treatment at the incubation time and concentrations tested. When cycloheximide incubation was extended to 4 h to match TRAIL assay conditions, a slight decrease in GAPDH was observed at high cycloheximide concentrations and cycloheximide appeared to be somewhat more potent (Fig 2B).

### Correlation of effects of protein synthesis inhibitors on TRAIL-induced apoptosis with protein synthesis inhibition by ICW assay

In order to demonstrate the applicability of the assay, several protein synthesis inhibitors were assessed for their ability to sensitize TRAIL-resistant ACHN cells to TRAIL-induced apoptosis in comparison with their ability to inhibit protein synthesis in the same cells. The TRAIL sensitization assay protocol [22] included a 4 h pretreatment with compounds before the addition of TRAIL. Therefore, the protein synthesis assay was applied both after 15 min (as in the assay development experiments) and after 4 h incubation with inhibitors. Reduction in protein synthesis after 4 h as measured by the ICW assay generally correlated with TRAIL-induced apoptosis in a dose-dependent manner (S3 and S4 Figs). IC_50_ values were estimated and summarized in Table 1. As expected for a TRAIL-resistant cell line, TRAIL alone had minimal growth inhibitory effects (> 90% cell survival by XTT assay).

**Table 1:** Effects of inhibitors on TRAIL-induced apoptosis and protein synthesis.

	IC_50_ (nM), ave ± se		
	TRAIL assay		
compound	- TRAIL	+ TRAIL	protein synthesis (puro signal), 4 h
anisomycin	113 ± 27	33.3 ± 0.2	219 ± 70
cycloheximide	>10,000	108 ± 25	358 ± 110
emetine	>1000	100 ±18	29.8 ± 4.8
glaucarubinone	>10,000	134 ± 13	481 ± 79
verrucarin A	ND^a^	1.4 ± 0.1	5.0 ± 0.4

^a^could not be estimated from dose-response curve

**Fig 3:**
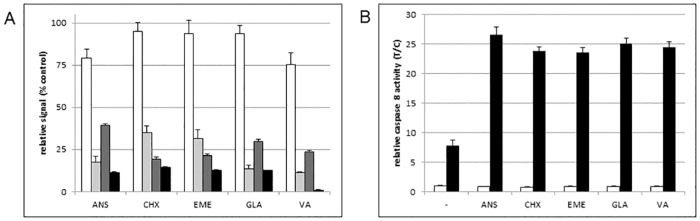
Effects of protein synthesis inhibitors on protein synthesis, levels of cFLIP protein, and TRAIL- induced apoptosis. (A) ACHN cells were treated for 4 h with 1 μM of the indicated compound at which point cells were processed for ICW detection and quantitation of GAPDH (open bars), puromycin (light grey), or cFLIP (dark grey). In a parallel experiment, TRAIL was added after 4 h with compounds and cell survival assessed after 24 h by the XTT assay (black bars). Signals were normalized to control on the same plate (vehicle control = 100%). Error bars represent sd (n = 4 for puromycin and GAPDH; n = 3 for cFLIP; and 3 plates, duplicate wells per plate for compounds + TRAIL). (B) Caspase 8 activity was measured after 4 h treatment with 1 μM of the indicated compound followed by 4 h in the absence (open bars) or presence (black bars) of TRAIL and normalized (vehicle control = 1.0). Error bars represent sd (n = 3). compounds: ANS: anisomycin, CHX: cycloheximide, EME: emetine, GLA: glaucarubinone, VA: verrucarin A).

Treatment of cells with verrucarin A appeared to induce growth inhibition in the absence of TRAIL (S3 Fig). Protein synthesis inhibition after longer treatment (*i*.*e*., 18–24 h) with verrucarin A was therefore further assessed. At 100 nM verrucarin A, the GAPDH signal was reduced to 56.2 ± 1.2% of control as compared to 47.8 ± 1.9% of control for total cell number suggesting that GAPDH may be a useful surrogate for cell numbers and may be appropriate for further normalization where cell growth data are unavailable. As noted (S3 Fig), this is consistent with a growth inhibitory effect rather than toxicity for verrucarin A at these concentrations and time points. Previous studies suggested that cycloheximide-induced sensitization of ACHN cells to TRAIL occurred as a result of loss of cFLIP protein [18]. Therefore, the effects of all 5 inhibitors on cFLIP levels were assessed (also in the ICW format). Fig 3A shows the relative GAPDH, puromycin, and cFLIP ICW signals after 4 h treatment along with relative cell survival (XTT) after 4 h compound followed by 24 h TRAIL. Puromycin and cFLIP ICW signals were markedly reduced by all 5 compounds as was cell survival in the presence of compound + TRAIL. In order to confirm that TRAIL sensitization actually occurred via TRAIL-dependent apoptotic signaling, TRAIL-dependent activation of caspase 8 (TRAIL death receptor-induced caspase) [11–15] was assessed after 4 h compound followed by 4 h ± TRAIL [22]. None of the compounds alone affected caspase 8. However, after TRAIL addition, very large increases in caspase 8 activity were observed in all cases (Fig 3B).

## Discussion

Utilization of puromycylation has been extensively validated as an approach for assessing protein synthesis [1–6, 23]. However, the low throughput nature of the applications described to date (cellular imaging, immunoblot/densitometry, etc.) [1–6, 23–27] would tend to limit its scope. The method described herein broadens the scope of potential applications of protein puromycylation to include high-throughput applications wherein larger numbers of samples would need to be quantitated in an *in situ* cell-based assay. Similarly, this method could easily be configured to correlate levels of protein synthesis to levels of or activation state of specific regulatory or signaling proteins in order to assess molecular targets for modulation of translation (*e*.*g*., by measuring a specific signaling protein or phosphoprotein rather than GAPDH or other “housekeeping” protein) or consequences of altering protein synthesis in normal cells and a variety of disease states. Puromycylation is able to measure protein synthesis in any cellular context and this method would be widely adaptable to multiple cell types and conditions as well as being significantly easier and less subjective for quantitative analysis than many alternative approaches. The antibodies used in this report are highly specific as demonstrated by standard western blot applications and there is quantitative correlation between westerns and ICW.

The choice of ACHN renal carcinoma cells was made in part because of their resistance to TRAIL-induced apoptosis, a phenomenon subject to high-throughput screening [22]. These cells can be sensitized by protein synthesis inhibition leading to decreased levels of the anti-apoptotic protein cFLIP [17, 18]. Clearly, an increased ability to probe protein synthesis inhibition may lead to important understanding of TRAIL-induced apoptosis as well as a variety of other phenomena affected by translation inhibitors. The inhibitors chosen for this study, anisomycin, cycloheximide, emetine, glaucarubinone, and verrucarin A, all clearly sensitize ACHN cells to TRAIL-induced apoptosis as measured both by TRAIL-dependent cell death and TRAIL-dependent caspase 8 activation (*i*.*e*., death receptor signaling) after pretreatment with the inhibitors. In parallel, each of the inhibitors also affects protein synthesis in the same cells. Interestingly, with the exception of emetine, they were generally less potent as protein synthesis inhibitors than as TRAIL sensitizers. Multiple explanations are possible for this observation. First, inhibition of protein synthesis may only need to reach a threshold level in order to sensitize the cells. Cellular levels of cFLIP are quantitatively controlled at the synthesis and degradation levels [28, 29] and overexpression of cFLIP is a common mechanism of TRAIL resistance. Protein synthesis inhibition by anisomycin [17] and cycloheximide [18] has been reported to sensitize resistant cells to TRAIL-induced apoptosis via downregulation of cFLIP. Effective reduction in levels of cFLIP protein may not require complete inhibition of its synthesis. The demonstration of significant, but not complete loss of cFLIP protein is consistent with this hypothesis. It is also possible that different isoforms of cFLIP contribute differentially to sensitization of cells to TRAIL [28, 29]. The antibody used here does not distinguish between isoforms. Although the ICW assay for cFLIP clearly shows loss of total cFLIP, further investigation would be required in order to implicate specific isoform(s) or to understand a possible threshold effect. Second, global reduction in protein synthesis by itself could lead to increased susceptibility to apoptotic stimuli [30, 31]. Finally, many protein synthesis inhibitors also have other cellular effects including induction of cellular stress phenomena [30–33] and activation of JNK [34] as well as a variety of other cellular effects. Thus, these compounds may be enhancing TRAIL signaling via mechanisms other than reduction in protein synthesis and/or they may also induce the intrinsic (mitochondrial) apoptotic pathway as reported for anisomycin [35], quassinoids [36], and verrucarin A [37] for example. Further application of the protein synthesis assay will allow for rapid quantitative analysis of this aspect of their activity. It is therefore vital to employ a reasonably high-throughput quantitative method for evaluation of protein synthesis inhibition (*e*.*g*., dose-, time-, cellular context-dependent conditions, etc.) in parallel with standard approaches for analysis of apoptotic signaling to provide useful insights into the effects of protein synthesis inhibitors in this context. The method described has several other advantages in addition to throughput. As discussed above, quantitation by ICW is clearly advantageous for puromycylation as compared to standard western blot in terms of signal definition and quantitation, clearly defined and minimal backgrounds, and reliability. Furthermore, as seen in the results with ACHN cells, protein synthesis can easily be assessed under exactly the same conditions (*e*.*g*. cell density/growth conditions, even identical assay plates if desired) as parallel investigation of other phenomena (in this case cell proliferation and apoptosis). Although not relevant to this report, it would also be possible to assess puromycylation of cell surface proteins by eliminating the ICW permeablilization step rather than by processing for FACS analysis. This could have advantages, particularly for adherent cells. The application discussed here is limited to ACHN cells and relevance to TRAIL sensitization, but the method would clearly have widespread potential applicability to virtually any cellular phenomena related to protein synthesis.

## Conclusions

The unique combination of puromycin labeling of nascent polypeptides in cells with the quantitative microplate-based ICW analysis of incorporated puromycin provides a powerful high-throughput, non-radioactive method for taking a quantitative snapshot of protein synthesis under various cellular conditions. As such, this assay is of clear utility for assessment of correlations between protein synthesis inhibition and cellular phenomena like (in the example shown here) TRAIL sensitization.

## Supporting Information

S1 Fig Signal/background for quantitation of puromycin and GAPDH (ICW format). (PDF)

S2 Fig Detection of signals in standard western blot format. (PDF)

S3 Fig Effects of protein synthesis inhibitors on ACHN cells. (PDF)

S4 Fig Dose-dependent effects of protein synthesis inhibitors on puromycylation and TRAIL-induced apoptosis. (PDF)

S1 File Quantitation and data quality parameters. (PDF)

S1 Table Effect of cycloheximide on puromycylation (standard western blot format). (PDF)
