# Supplementary material for: A Microplate-Based Nonradioactive Protein Synthesis Assay: Application to TRAIL Sensitization by Protein Synthesis Inhibitors
Source: PLoS One. 2016 Oct 21;11(10):e0165192. doi: 10.1371/journal.pone.0165192 (PMC5074477; doi:10.1371/journal.pone.0165192)
Supplement: S1 Fig — (PDF) [file pone.0165192.s001.pdf]

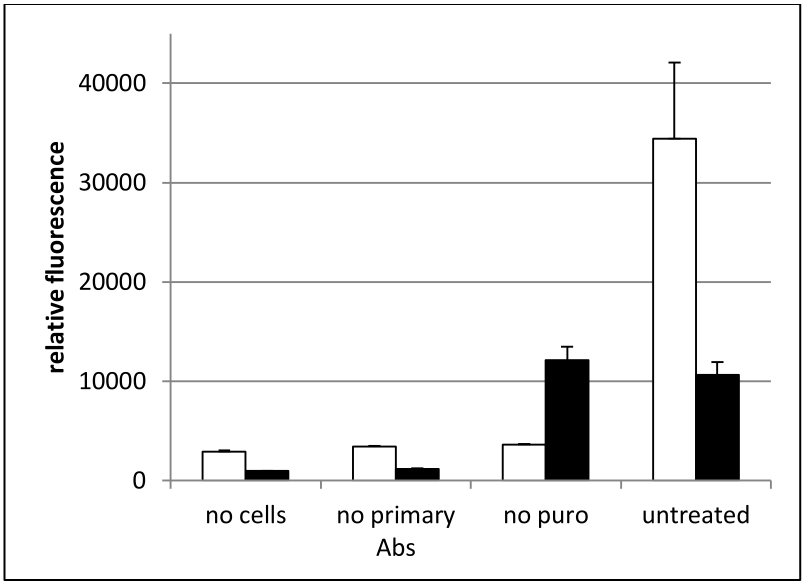

S1 Fig. Signal/background for quantitation of puromycin and GAPDH (ICW format). Relative fluorescence values for control wells from a representative ICW plate are based on raw data from the scanner. Open bars: puromycin (red) signal; black bars, GAPDH (green) signal ± sd. "Untreated" represents puromycin-labeled cells.

S/B averaged 11.2 for red (puromycin) signal and 8.2 for green (GAPDH) signal.
