# Supplementary material for: A Microplate-Based Nonradioactive Protein Synthesis Assay: Application to TRAIL Sensitization by Protein Synthesis Inhibitors
Source: PLoS One. 2016 Oct 21;11(10):e0165192. doi: 10.1371/journal.pone.0165192 (PMC5074477; doi:10.1371/journal.pone.0165192)
Supplement: S2 Fig — (PDF) [file pone.0165192.s002.pdf]

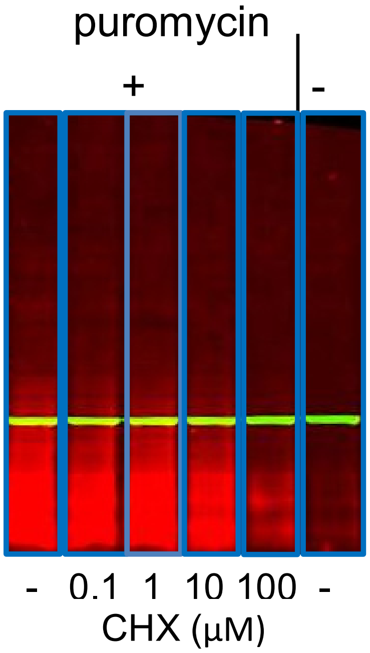

S2 Fig. Detection of signals in standard western blot format. ACHN cells were pretreated with CHX for 15 min followed by puromycin labeling and western blot analysis. Rectangles represent lanes for quantitation of the red (puromycin) signal.

Quantitation of puromycin lanes: Lane 6 (puromycin -) was defined as background and its fluorescence background was subtracted from the signals in each of the other lanes giving the following results:

Signal to background (S/B) = lane 1 (untreated sample)/lane 6 = 1.5. This is in marked contrast to the S/B ratio of 8-11 calculated for ICW (S1 Fig).

Quantitation of the GAPDH (green band) is straightforward with scanning software – simply select the band and record fluorescence. However, selection of entire lanes can be considerably more complex particularly since the areas should be identical in size.
