# Supplementary material for: A Microplate-Based Nonradioactive Protein Synthesis Assay: Application to TRAIL Sensitization by Protein Synthesis Inhibitors
Source: PLoS One. 2016 Oct 21;11(10):e0165192. doi: 10.1371/journal.pone.0165192 (PMC5074477; doi:10.1371/journal.pone.0165192)
Supplement: S3 Fig — (PDF) [file pone.0165192.s003.pdf]

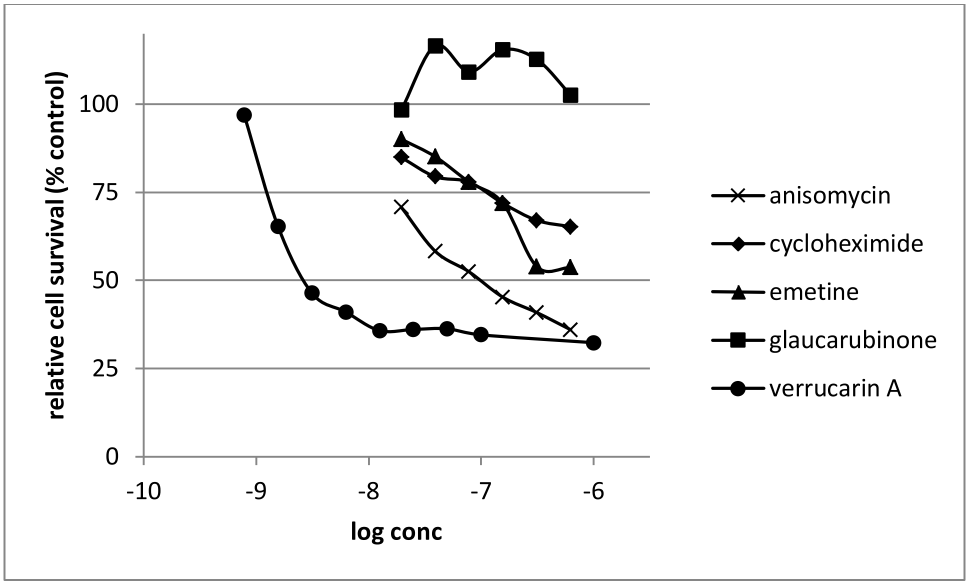

S3 Fig. Effects of protein synthesis inhibitors on ACHN cells. ACHN cells were treated for 24 h with the indicated compound and relative cell suvival were assessed by the XTT assay (untreated control = 100%). Error bars were left off for clarity.

As individual agents, the effects varied from innocuous (gluacarubinone) to significant apparent growth inhibition toxicity and/or growth inhibition. Verrucarin A was particularly interesting in that above ~10 nM, it no longer has a dose-dependent effect (as would be expected for cell killing). Given that the doubling time for these cells is 27.5 h (per NCI documentation: https://dtp.cancer.gov/discovery_development/nci-60/cell_list.htm), a flat curve bottoming out at 30-40% of control like this is probably reflective of growth inhibition rather than toxicity. The mechanism(s) of growth inhibition vs. cytotoxicity are beyond the scope of this project, but the results suggest differential phenomena contributing to each. The lack of effect of glaucarubinone in the absence of TRAIL suggests that protein synthesis inhibition *per se* may not be toxic in the time scales and concentrations used in this study. The data from this experiment were used to generate IC_{50} values (where possible) for inclusion in Table 1 in the main text.
