# Supplementary material for: A Microplate-Based Nonradioactive Protein Synthesis Assay: Application to TRAIL Sensitization by Protein Synthesis Inhibitors
Source: PLoS One. 2016 Oct 21;11(10):e0165192. doi: 10.1371/journal.pone.0165192 (PMC5074477; doi:10.1371/journal.pone.0165192)
Supplement: S4 Fig — (PDF) [file pone.0165192.s004.pdf]

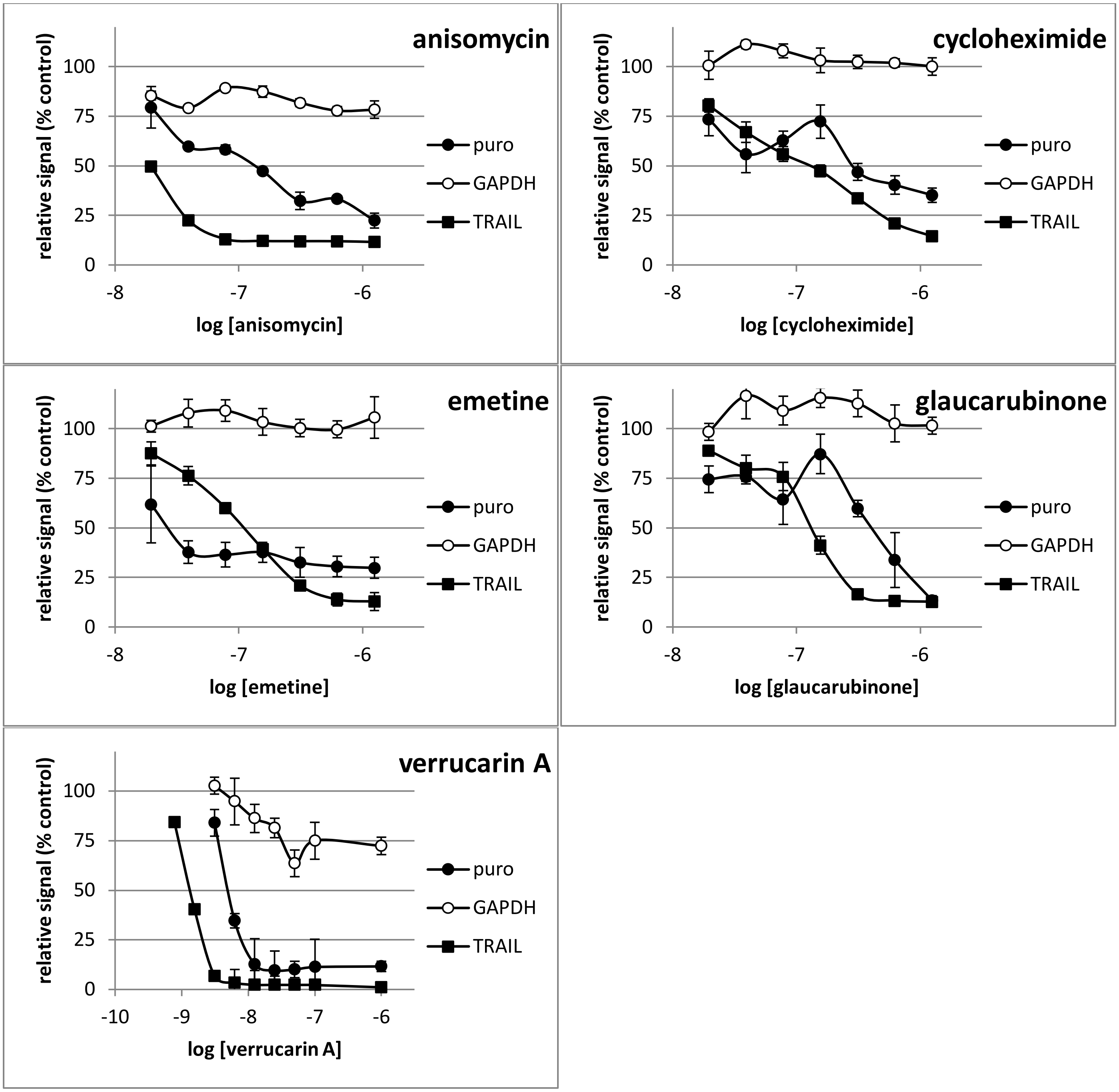

S4 Fig. Dose-dependent effects of protein synthesis inhibitors on puromycylation and TRAIL-induced apoptosis. ACHN cells were treated for 4 h with the indicated compound followed by ICW assay for puromycylation (puro), control protein (GAPDH). After an additional 24 h treatment with TRAIL, cell survival was assessed (XTT assay). untreated = 100%. Error bars represent sd (n = 4). The data from this experiment were used for calculation of IC_{50} values as discussed in the text and summarized in Table 1.
