# Supplementary material for: A Microplate-Based Nonradioactive Protein Synthesis Assay: Application to TRAIL Sensitization by Protein Synthesis Inhibitors
Source: PLoS One. 2016 Oct 21;11(10):e0165192. doi: 10.1371/journal.pone.0165192 (PMC5074477; doi:10.1371/journal.pone.0165192)
Supplement: S1 File — (PDF) [file pone.0165192.s005.pdf]

S1 File. Quantitation and assay quality parameters.

Signal to background

S/B can be calculated in the InCell western (ICW) experiments using a number of definitions of "background." Controls included 1) wells without cells, but otherwise treated identically to experimental, 2) puromycin-labeled cells processed in the absence of primary antibodies, and 3) wells containing cells that were not labeled with puromycin. S1 Fig shows an example of these three types of controls (using raw values from the Odyssey scanner) in comparison to high control values (*i.e.*, puromycin-labeled, but otherwise untreated cells). For subsequent analysis, background was defined as no cell control wells.

Given that puromycin labeling of proteins followed by western blot detection results in a smear of signal down an electrophoretic lane (as seen in Figure 1 in the main text, S2 Fig and references associated with puromycin labeling), the challenge of quantitating results in a western blot format are significant. Along with issues with regard to defining lanes and ensuring equal areas for quantitation, it is clear from the results in S1 Table that general background is somewhat higher in blots given that the fluorescence associated with control lanes in a blot are relatively much higher than those in an ICW plate.

Z' calculation (ICW only):

Although high-throughput applications (*e.g.*, HTS) were not included in this report, one of the significant advantages of the ICW is the ability to assess large numbers of samples. A widely used assessment of assay reproducibility in a high-throughput application is the Z' factor calculation (Zhang, et al., 1999).

$Z' = 1 - [3 \times (sd_{\text{high control}} + sd_{\text{low control}})/(\text{average value}_{\text{high control}} - \text{average value}_{\text{low control}})]$

Controls are the same as for S/B calculations (high control = puromycin-labeled untreated cells, low control = no cell wells). Z' averaged 0.50 for puromycin and 0.75 for GAPDH, both values reflective of a reliable high-throughput assay.

Zhang J-H, Chung TDY, Oldenburg KR: A simple statistical parameter for use in evaluation and validation of high throughput screening assays. *J. Biomol. Screen* 1999;4:67-73.
