# Supplementary material for: A Microplate-Based Nonradioactive Protein Synthesis Assay: Application to TRAIL Sensitization by Protein Synthesis Inhibitors
Source: PLoS One. 2016 Oct 21;11(10):e0165192. doi: 10.1371/journal.pone.0165192 (PMC5074477; doi:10.1371/journal.pone.0165192)
Supplement: S1 Table — (PDF) [file pone.0165192.s006.pdf]

S1 Table. Effect of cycloheximide on puromycylation (standard western blot format).

[CHX], μM	Relative fluor (- bkg), pixels	T/C
0	26970896	1.00
0.01	23247008	0.86
0.1	19150016	0.71
1	9441088	0.35
10	5430528	0.20
